# Hemophagocytosis lymphocytosis presenting as pulmonary‐renal syndrome: a case report and literature review

**DOI:** 10.1002/ccr3.1082

**Published:** 2017-07-27

**Authors:** Morgan Wong, Arpit Rao, Jacklyn Nemunaitis, David Czuchlewski, Shazib Sagheer, Cecilia Arana‐Yi

**Affiliations:** ^1^ Department of Internal Medicine University of New Mexico MSC10‐5550 Albuquerque 87131 New Mexico; ^2^ Division of Hematology and Oncology Department of Internal Medicine University of New Mexico Comprehensive Cancer Center MSC07‐4025 Albuquerque 87131 New Mexico; ^3^ Department of Pathology University of New Mexico MSC10‐5550 Albuquerque 87131 New Mexico

**Keywords:** Hemophagocytosis lymphocytosis, pulmonary‐renal

## Abstract

Hemophagocytosis Lymphocytosis (HLH) is a rare and life‐threatening illness that is more commonly seen in infants; however, its incidence in adults is becoming more common. Recognizing HLH in a complicated clinical scenario is key to early recognition, treatment, as well as improved morbidity and mortality.

## Introduction

Hemophagocytosis Lymphocytosis (HLH) is a rare condition characterized by a dysregulated and hyperactive immune response. HLH is more common in infants, but the incidence is increasing in adults with increasing awareness. HLH can affect any organ, and pulmonary or renal involvement is uncommon, but associated with worse outcomes including increased in‐hospital and 6‐month mortality. We describe the case of secondary HLH presenting as pulmonary‐renal syndrome.

## Case

A 56‐year‐old man was admitted to the medical intensive care unit for acute hypoxemic respiratory failure. Initial chest X‐ray showed bilateral extensive multifocal nodular pulmonary opacities. He had a temperature of 38.9°C, blood pressure of 82/54 mmHg, heart rate of 51 per min, and oxygen saturation of 91% on supplemental O2 (FiO2 of 95%) on admission.

For suspected severe sepsis from healthcare‐associated pneumonia, he was started on vancomycin, piperacillin‐tazobactam, and methylprednisolone 50 mg IV every 8 h. Laboratories showed pancytopenia with white blood cell (WBC) count of 1.6 × 10^3^/*μ*L, absolute neutrophil count (ANC) of 0.7 × 10^3^/*μ*L, hemoglobin of 11.3 g/dL, hematocrit of 36%, and platelet count of 99 × 10^3^/*μ*L. Creatinine was elevated at 1.07 mg/dL from a baseline of 0.38 mg/dL. Liver function tests were within normal range except for albumin of 1.8 g/dL. Urinalysis showed new onset microscopic hematuria and nephritic‐range proteinuria (70.2 mg/dL). Serum ferritin was elevated at 7512 ng/mL (normal 26–388 ng/mL). Abdominal ultrasound showed splenomegaly (spleen size was 17.7 cm). Hematology, Rheumatology, and Nephrology services were consulted, but a renal biopsy was unable to be done due to critical status.

## Prior History

**Figure 1:**
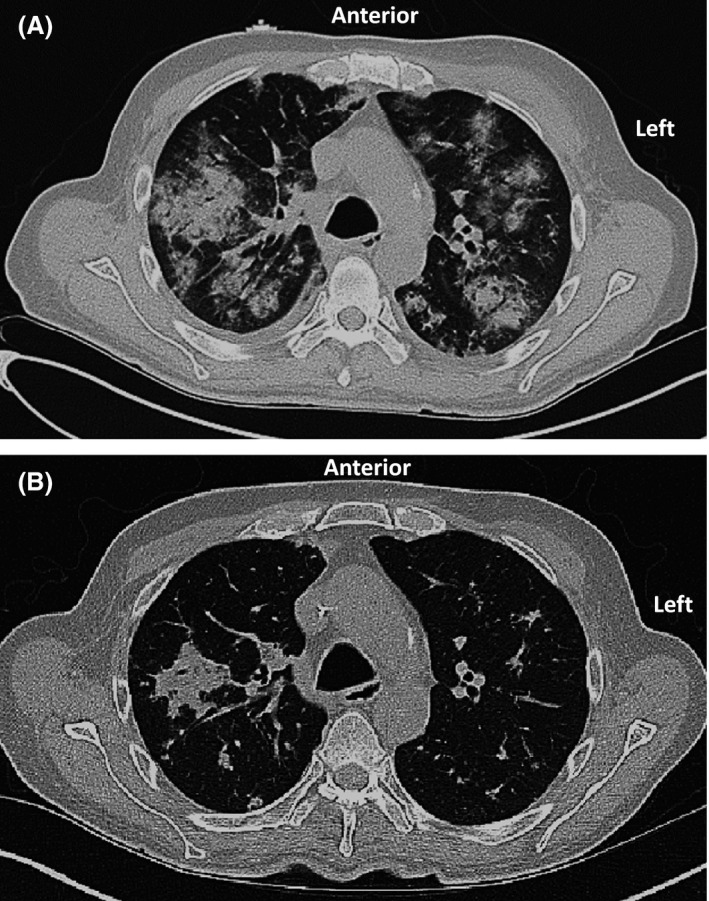
(A) A CT scan at the time of hospitalization showing scattered bilateral pulmonary nodules with cavitary lesions. (B) A CT scan after 3 weeks of therapy (per HLH‐94 protocol) showing remarkable improvement in bilateral pulmonary nodular infiltrates.

The patient narrated a history of worsening pulmonary symptoms including shortness of breath starting approximately a year prior to the current hospitalization. The patient had three prior admissions for acute hypoxemic respiratory failure since symptom onset, and an extensive workup had failed to reveal an underlying cause. Prior workup included several computed tomography (CT) scans of the chest that showed scattered pulmonary nodules and cavitary lesions (Fig. 1A and B). Four lung biopsies and a video‐assisted‐thoracic surgery (VATS) with wedge pulmonary resection were performed, but pathology only showed scattered fibroplastic foci, chronic inflammation, and necrotizing debris without signs of malignancy. A bronchoscopy performed a month before the current admission showed normal tracheobronchial tree. The concurrent bronchoalveolar lavage culture grew pan‐sensitive *Pseudomonas aeruginosa*, which was treated with a complete course of antimicrobials. This only partially improved patient's respiratory status.

The patient also suffered from an episode of diffuse erythematous maculopapular rash on his trunk, groin, and face, and associated oral mucosal ulcers a few months prior to current presentation. Punch biopsy of a skin lesion revealed superficial and deep lymphocytic infiltrate with vacuolar interface change.

Finally, while an exhaustive autoimmune workup was negative, polyangiitis granulomatosis remained a possible differential diagnosis due to pulmonary and renal involvement. Hence, the patient was given at least 2 weeks of high‐dose prednisone with taper based by the Rheumatology team, which resulted in some improvement in symptoms.

## Workup and Management During Current Admission

**Figure 2:**
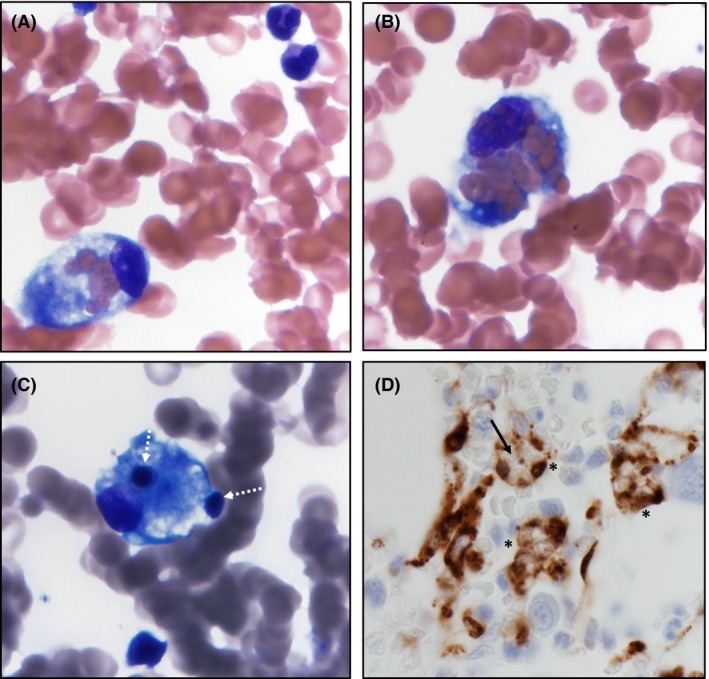
(A and B) (Wright stain, 1000x) show a single histiocyte performing hemophagocytosis with ingestion of mature RBCs. (C) (Wright stain, 1000x), the histiocyte is seen ingesting immature erythroid precursors (white dotted arrows). (D) (IHC for CD68, 600x) shows several CD68 positive histiocytes (*) with “negative” images of the ingested cells (black arrow) within them.

Our team suspected a diagnosis of HLH and performed a bone marrow biopsy, which showed extensive hemophagocytosis in the marrow (Fig. 2A–D). Patient met six of eight HLH‐94 criteria necessary for diagnosis (including fever greater than 100.4 for more than 7 days, cytopenia, splenomegaly, hypofibrinogenemia, elevated ferritin, elevated triglycerides, and hemophagocytosis in bone marrow.) Further viral and infectious causes were ruled out with negative EBV, CMV, Hepatitis, and HIV screening, as well as negative blood and urine cultures from this current admission. Given the pulmonary and renal involvement, our final diagnosis became secondary HLH with pulmonary‐renal syndrome.

The patient was emergently started on treatment per the HLH‐94 protocol and received dexamethasone 10 mg/m^2^ IV daily, and etoposide 150 mg/m^2^ IV twice weekly. A magnetic resonance imaging (MRI) scan of the brain showed cytotoxic edema in the anterior and lateral frontal lobes. Lumbar puncture showed elevated cerebral spinal glucose level of 124 mg/dL. Intrathecal methotrexate (15 mg dose) was empirically administered during the procedure given the high probability of CNS involvement by HLH.

Patient's clinical status including respiratory status and serum ferritin began to improve within hours of initiation of therapy. Ferritin levels had a fourfold reduction within 1 week, and patient was discharge home after 2 weeks without a need for supplemental oxygen. Urine microhematuria and proteinuria improved as well. Three months after diagnosis, patient has continued to have a remarkable improvement in status, and is tolerating the treatment very well.

## Discussion

Hemophagocytosis Lymphocytosis is a life‐threatening illness that is rarely seen in adulthood. Epidemiologic studies in Japan report the incidence rate at 1 per 800,000 in adults with a median age of 50 years and a male predominance 1, 2. Published studies note a median survival time of 1.8–2.2 months, and an overall mortality of 42.2% 3.

Presentation can be diverse, and the primary etiology is unable to be identified in as many as 18% of cases 4. Incidence of pulmonary or renal involvement has been reported to be as high as 55–60% in two institutional case series 3, 5. In one of these studies, 54% of 219 patients in a national HLH reference noted pulmonary involvement due to infection 44%, pulmonary edema 28%, and undetermined causes 23%. Pulmonary involvement further carried a 52.5% in‐hospital mortality risk compared with 20% for those without pulmonary involvement 3. Another 95‐patient single‐institution study noted that 62% of patients presenting with HLH had concurrent renal involvement and met criteria for Acute Kidney Injury (AKI). This study further noted an increase in 6‐month mortality with stage 2 or greater AKI 6.

A case series of patients in the intensive care unit reported the presence of multiple organ failure in 59% of the patients with confirmed diagnosis of HLH. Mechanical ventilation and renal replacement therapy were required in 75 and 55% of these patients, respectively 5. Finally, only one case of granulomatosis polyangitis associated with HLH has been described 7.

Uniquely in our case, this patient's HLH demonstrated pulmonary and renal involvement without a unifying underlying primary diagnosis other than HLH. He also had a protracted clinical course with a period of 1 year between symptom onset and HLH diagnosis, which is incredibly rare, and may signify a rare indolent form of HLH. The ongoing genetic workup may help delineate a cause and identify a novel group of HLH patients.

Given the initially atypical findings, this case also presents a learning opportunity for clinicians, as such a complicated clinical scenario could be mistaken for other nonhematologic etiologies, and a delay in diagnosis is very likely to be fatal given the rapidly progressive nature of HLH. Based on the experience from our institution, we recommend including HLH on the initial differential for such a presentation when preliminary workup is negative.

## Conflict of Interest

None declared.

## Authorship

MW: collaborated in conceiving, writing, and editing case study. AR: collaborated in conceiving, writing and editing case study. JN: collaborated in conceiving, writing and editing case study. DC: collaborated in obtaining and interpreting biopsy images as well as editing case study. SS: collaborated patient care, as well as writing and editing case study. CA‐Y: collaborated in conceiving, writing and editing case study.
